# Resistance to immune checkpoint inhibitors in colorectal cancer with deficient mismatch repair/microsatellite instability: misdiagnosis, pseudoprogression and/or tumor heterogeneity?

**DOI:** 10.37349/etat.2024.00231

**Published:** 2024-05-23

**Authors:** Nicola Normanno, Vincenza Caridi, Matteo Fassan, Antonio Avallone, Fortunato Ciardiello, Carmine Pinto

**Affiliations:** University of Barcelona, Spain; ^1^IRCCS Istituto Romagnolo per lo Studio dei Tumori (IRST) “Dino Amadori”, 47014 Meldola, Italy; ^2^Cell Biology and Biotherapy Unit, Istituto Nazionale Tumori-IRCCS-Fondazione G. Pascale, 80131 Naples, Italy; ^3^Department of Medicine (DIMED), Surgical Pathology Unit, University of Padua, 35100 Padua, Italy; ^4^Veneto Institute of Oncology, IOV-IRCCS, 35100 Padua, Italy; ^5^Medical Oncology, Istituto Nazionale Tumori-IRCCS-Fondazione G. Pascale, 80131 Naples, Italy; ^6^Department of Precision Medicine, The University of Campania Luigi Vanvitelli, 80138 Naples, Italy; ^7^Medical Oncology, Comprehensive Cancer Centre IRCCS-AUSL Reggio Emilia, 42121 Reggio Emilia, Italy

**Keywords:** Colorectal carcinoma, deficient mismatch repair, microsatellite instability, immune checkpoint inhibitors, resistance, tumor heterogeneity

## Abstract

Colorectal carcinoma (CRC) with deficiency of the deficient mismatch repair (dMMR) pathway/microsatellite instability (MSI) is characterized by a high mutation load and infiltration of immune cells in the tumor microenvironment. In agreement with these findings, clinical trials have demonstrated a significant activity of immune checkpoint inhibitors (ICIs) in dMMR/MSI metastatic CRC (mCRC) patients and, more recently, in CRC patients with early disease undergoing neoadjuvant therapy. However, despite high response rates and durable clinical benefits, a fraction of mCRC patients, up to 30%, showed progressive disease when treated with single agent anti-programmed cell death 1 (PD-1) antibody. This article discusses the three main causes that have been associated with early progression of dMMR/MSI mCRC patients while on treatment with ICIs, i.e., misdiagnosis, pseudoprogression and tumor heterogeneity. While pseudoprogression probably does not play a relevant role, data from clinical studies demonstrate that some dMMR/MSI CRC cases with rapid progression on ICIs may be misdiagnosed, underlining the importance of correct diagnostics. More importantly, evidence suggests that dMMR/MSI mCRC is a heterogeneous group of tumors with different sensitivity to ICIs. Therefore, we propose novel diagnostic and therapeutic strategies to improve the outcome of dMMR/MSI CRC patients.

## Mismatch repair pathway in the pathogenesis of colorectal carcinoma and response to immunotherapy

The mismatch repair (MMR) is a DNA repair pathway involved in maintaining genomic integrity and stability. This pathway plays a pivotal role in repairing DNA errors generated during replication, such as DNA base substitution mismatch, frameshift (insertion/deletion), and slippage [1]. The MMR recognizes and repairs DNA damage through protein complexes that involve among the others mutS homologue 2 (MSH2), MSH6, postmeiotic segregation increased 2 (PMS2), and mutL homologue 1 (MLH1). These proteins are arranged in heterodimeric complexes that bind the areas of abnormal DNA and initiate its removal.

A deficiency of the MMR pathway [deficient MMR (dMMR)] can be derived from germline and/or somatic mutations or epigenetic silencing (e.g., hypermethylation of *MLH1* promoter or secondary epigenetic silencing of *MSH6*) [2]. Germline pathogenic mutations of *MLH1*, *MSH2*, *MSH6* and *PMS2* cause the Lynch syndrome, the most common type of hereditary cancer syndrome affecting 1 in 280–400 individuals [3].

In neoplastic cells, the dMMR leads to an hypermutated phenotype with accumulation of gene mutations, mainly small insertions/deletions producing a high tumor mutational burden (TMB) [4]. The dMMR status determines also an altered length of microsatellites, repeated sequences of 1–6 nucleotides widely distributed in the genome, that is referred to as microsatellite instability (MSI) [5]. MSI is therefore a consequence of dMMR.

Colorectal carcinoma (CRC) is a molecularly heterogeneous disease whose pathogenesis involves different mechanisms. Approximately 15% of CRC show a dMMR/MSI phenotype. The majority of dMMR/MSI CRC are sporadic and are due to hypermethylation of the MLH1 promoter (12%). Germline mutations of genes involved in the MMR pathway and leading to the Lynch syndrome occur in approximately 3% of CRC. The concomitant presence of a *BRAF* V600E mutation occurs in about 30% of cases and is limited to sporadic dMMR/MSI [6].

The frequency of dMMR/MSI varies according to the stage of disease. A higher incidence is found in the early CRC stages (about 20% in stage I and II, and 13% in stage III), while a lower incidence is observed in the metastatic setting (4–5% in stage IV) [7]. Such difference is due to the favorable prognosis of dMMR/MSI CRC patients, who indeed do not benefit from adjuvant treatment with 5-fluorouracil (5FU) in stage II [8]. Only a small fraction of dMMR/MSI CRC has a recurrence of the disease after surgery or are diagnosed in advanced stage. Interestingly, dMMR/MSI metastatic CRC (mCRC) patients show a worse prognosis as compared with microsatellite stable (MSS) mCRC [9, 10]. In particular, dMMR/MSI mCRC is resistant to conventional therapeutic regimens, although some studies revealed a favorable effect of regimens with irinotecan and bevacizumab in this subtype [11, 12]. Although only 4% of mCRCs are dMMR/MSI, they nevertheless represent a relevant population, the expected cases of CRC being 1.9 million in 2020, with a fraction of over 20% metastatic at diagnosis and up to 50% that may develop metastases in time [13].

The gene expression profiling-based classification of CRC in consensus molecular subtypes (CMS), confirmed the presence of most dMMR/MSI tumors in the CMS1 subgroup that is characterized by a high mutation load and infiltration of immune cells in the tumor microenvironment (TME) [14]. The high TMB of dMMR/MSI CRC leads to the synthesis of aberrant and potentially immunogenic neoantigens by tumor cells that, after the presentation on the major histocompatibility complex (MHC) of antigen-presenting cells (APCs), are recognized as foreign by T cells [15]. As a consequence, dMMR/MSI tumors are heavily infiltrated with activated cytotoxic T lymphocytes (CTLs) and helper T cell 1 (Th1) cells [16]. During cancer progression, to counterbalance the antitumor Th1/CTLs immune response, dMMR/MSI cancer cells upregulate the expression of multiple active immune-checkpoints (ICKs) such as programmed cell death 1 (PD-1), programmed cell death ligand 1 (PD-L1), and cytotoxic T-lymphocyte antigen 4 (CTLA-4) causing functional exhaustion and a lack of response by tumor-infiltrating lymphocytes (TILs). Therefore, ICKs expression would protect highly immunogenic tumor cells from being destroyed by CD8^+^ T cells, which lose their anticancer effector functions. Based on these findings, the use of immune checkpoint inhibitors (ICIs) appears as a suitable strategy to increase the antitumor immune response in patients with dMMR/MSI mCRC [16].

This hypothesis has been confirmed in several clinical trials that demonstrated a significant activity of ICIs in dMMR/MSI CRC. In the CheckMate-142 phase II study of nivolumab in 74 previously treated dMMR/MSI mCRC, an objective response rate of 31.1% was observed [17]. A phase II randomized trial of avelumab *versus* chemotherapy with- or without targeted therapy in 122 dMMR/MSI mCRC patients who received a previous line of therapy also showed the superiority of avelumab with respect to progression-free survival (PFS), with estimated percentages of patients alive and progression free at 12 months of 31.2% in the avelumab arm and 19.4% in the chemotherapy arm [18]. In the Keynote-177 phase III trial, pembrolizumab first-line therapy led to a statistically significant longer PFS as compared with standard chemotherapy in 307 dMMR/MSI mCRC patients [hazard ratio (HR) 0.60; *P* = 0.0002] [19]. The combination of ipilimumab plus nivolumab showed an overall response rate (ORR) of 62% in a phase II trial of first-line therapy in 45 dMMR/MSI mCRC [20].

The high sensitivity of dMMR/MSI CRC to ICI has been confirmed by recent data from clinical trials of neoadjuvant immunotherapy for early disease, showing a high pathological complete response (pCR) rate: NICHE I (20 patients treated with ipilimumab and nivolumab, with or without celecoxib; pCR 69%) [21]; NICHE II (112 patients receiving ipilimumab and nivolumab; pCR 67%) [22]; NICHE III (19 patients treated with nivolumab + relatlimab; pCR 79%) [23]; NCT0482572 (35 patients treated with pembrolizumab; pCR 65%) [24]; and NCT04165772 (12 patients with locally advanced rectal cancer receiving dostarlimab; pCR 100%) [25].

Based on available data, the PD-1 inhibitors, pembrolizumab and nivolumab, have been approved by the US Food and Drug Administration to treat patients with dMMR/MSI mCRC, while the European Medicine Agency approved pembrolizumab for the same indication.

However, despite high response rates and durable clinical benefits, a fraction of patients, up to 30%, showed progressive disease when treated with single agent anti-PD-1 antibody [19]. These findings were confirmed in other studies including the randomized phase II clinical trial of avelumab *versus* standard second-line chemotherapy in MSI CRC [18].

In this brief article, we will discuss the three main causes that have been associated with early progression of dMMR/MSI mCRC patients while on treatment with checkpoint inhibitors, i.e., misdiagnosis, pseudoprogression and tumor heterogeneity, in order to propose novel diagnostic and therapeutic strategies to improve the outcome of this subgroup of patients.

## Misdiagnosis

Immunohistochemistry (IHC) and polymerase chain reaction (PCR) are the most commonly used methods to identify dMMR/MSI tumors [26]. IHC detects the MMR protein loss of expression in formalin fixed paraffin embedded (FFPE) histologic specimens using antibodies that recognize the main proteins involved in dMMR, i.e., MLH1, PMS2, MSH2, and MSH6 (Table 1).

**Table 1:** Methods for dMMR/MSI testing

**Method**	**Characteristics**
Immunohistochemistry (IHC)	Detects loss of expression of MMR proteins in tumor specimens Uses antibodies directed against MLH1, PMS2, MSH2, MSH6 Staining pattern useful for screening of the Lynch syndrome
Polymerase chain reaction (PCR)	Detects the altered length of microsatellite loci as consequence of functional failure of MMR proteins Available reference panels use different mononucleotide and/or dinucleotide markers Usually, an MSI phenotype is defined by the presence of at least two unstable markers compared with healthy tissue or at least three unstable markers in the absence of healthy tissue
Next generation sequencing (NGS)	MSI usually determined by analyzing a large number of DNA markers Less standardized as compared with IHC and PCR

MMR: mismatch repair; dMMR: deficient MMR; MLH1: mutL homologue 1; PMS2: postmeiotic segregation increased 2; MSH2: mutS homologue 2; MSI: microsatellite instability; IHC: immunohistochemistry

MMR proteins act as functional heterodimers. The heterodimer MSH2/MSH6 can detect DNA mismatch alterations, whereas MLH1/PMS2 are key players in the repairing of the DNA sequence integrity. In these two heterodimers one of the proteins is the main partner (i.e., MLH1 and MSH2) and cannot be substituted by another component of the MMR complex. This implies that MLH1 and MSH2 silencing is usually associated with loss of PMS2 and MSH6, respectively. On the other hand, secondary partners (i.e., PMS2 and MSH6) when altered are substituted by alternative members of the MMR complex, maintaining the expression of the principal components. Tumors with isolated loss of MSH6 or PMS2 expression have a higher prevalence of germline mutations of the corresponding genes, whereas the most important mechanism of *MLH1* gene silencing is promoter hypermethylation.

The PCR is used to analyze the length of microsatellite loci, which are altered as consequence of functional failure of MMR proteins. PCR panels for MSI testing use mononucleotide and/or dinucleotide markers. Tumors are classified as MSI in presence of at least two unstable markers compared with healthy tissue or at least three unstable markers when healthy tissue is not available [27]. The concordance of IHC and PCR has been reported in the range of 90% to 97%. Next generation sequencing (NGS) can be also used for MSI detection, although this approach is less standardized.

Discordant results between local and centralized dMMR/MSI testing have been described in different studies. In the CheckMate-142 study of nivolumab in dMMR/MSI mCRC, tumor MMR/MSI status was locally assessed by IHC or PCR, while PCR was subsequently performed in a central laboratory on mandatory fresh tumor biopsies obtained before treatment [17]. Among 67 patients with available tissue for central testing, 53 (79.1%) were confirmed having MSI tumors while 14 (20.9%) were found to be MSS. Ten discordant cases were assessed locally by IHC and 4 with PCR. It must be emphasized that local and central testing were performed on tissue specimens collected at different time points.

In a retrospective analysis of two cohorts of mCRC classified as dMMR/MSI on the basis of local testing, central analysis found that 8% to 10% of the tumors were MSS [28]. Interestingly, 3/5 mCRC patients with rapid progression of the disease on ICI therapy were reclassified as MSS by central testing.

A large series of more than 3,000 cases confirmed a high degree of concordance between PCR/MSI and IHC/MMR tests, with only 1.6% of cases resulting discordant between the two tests [29]. Most of the discordant cases were due to IHC misclassifications. The authors concluded that discordant cases must be reviewed, and if needed, tests must be repeated and analyzed by an expert team. Pre-analytical and analytical factors affecting MMR testing should be considered and carefully evaluated in the clinical practice.

These data suggest that misdiagnosis of dMMR/MSI may be one of the causes of primary resistance observed in patients treated with ICI. Based on these findings, it is suggested to confirm the dMMR/MSI status with an orthogonal method before starting treatment with ICIs of a patient with mCRC, especially if the local test was not performed with a validated technique and in a certified laboratory.

## Pseudoprogression

Pseudoprogression is an unusual response pattern that has been observed in patients treated with immunotherapy [30, 31]. In particular, pseudoprogression is characterized by an initial increase in tumor size or even the appearance of new lesions, followed by a decrease in tumor volume. The frequency of pseudoprogression has been described to range between 3% and 10% in different studies. Such discrepancy is also due to the different criteria used in clinical trials to define this phenomenon. In a meta-analysis of 17 studies with 3,402 patients, the pooled incidence of pseudoprogression was 6% [32].

Clinical and radiological criteria can be used to differentiate pseudoprogression from true progression in patients receiving ICIs, including the use of specific criteria to assess response to immune therapy [30, 31]. In this respect, the ability of clinicians to recognize pseudoprogression significantly improved over the time [33]. In fact, most pseudoprogression occur in the first 3 months of treatment with ICI, and this phenomenon is often associated with improvement of symptoms and reduction in the levels of serum biomarkers [34]. The use of circulating tumor DNA (ctDNA) testing could greatly aid in the differential diagnosis of pseudoprogression *versus* true progression [35]. Indeed, in a study including 125 metastatic melanoma patients receiving anti-PD-1 antibodies, all patients (*n* = 9) with pseudo-progression showed a “favorable” ctDNA profile (i.e., ctDNA not detected at any time during treatment, or detectable at baseline and undetectable after treatment or decreased by at least 10-fold during treatment) [36]. In contrast, 18/20 patients with true progression of the disease had ctDNA detectable before and during treatment. Actually, the dynamics of ctDNA after treatment with immunotherapy showed to be strongly linked to the response and in general to the activity of the treatments in several trials of ICI in different tumor types [37, 38].

Overall, it is unlikely that in clinical trials of ICI in dMMR/MSI mCRC pseudoprogression might have played a significant role determining the interruption of a very active treatment.

## Tumor heterogeneity (true primary resistance): biological characteristics of the tumor cells and of the tumor immune microenvironment

The dMMR/MSI CRC subgroup has long been considered as a single entity, caused by a specific molecular mechanism that determines peculiar genomic alterations. However, increasing evidence suggests that this group of tumors is highly heterogeneous. For example, differences have been reported also within patients with germline variants linked to the Lynch syndrome, with *MSH6* mutant tumors showing lower levels of MSI and increased rate of single nucleotide variants, rather than indels [3].

Heterogeneity of dMMR/MSI CRC has been demonstrated at different levels. Loupakis et al. [39] reported the case of a mCRC patient with immunohistochemical and molecular heterogeneity in the primary tumor. The patient was treated with nivolumab plus ipilimumab and achieved a deep and lasting response with clear clinical benefit.

Marisa et al. [16] assessed the expression of immune checkpoint, tumor-infiltrating CTLs, cytotoxicity-related genes and metagenes (i.e., groups of genes with correlated expression), including immunoscore-like metagenes, in a large series of MSI and MSS CRC. The expression of immune checkpoint metagene was associated with worse prognosis in both early and metastatic MSI tumors, and showed correlation with reduced proliferation of infiltrating CD8^+^ T cells. Importantly, immune checkpoint metagene expression varied significantly among MSI CRC, leading to the identification of three groups, with high, intermediate and low expression. A correlation was found between high immune checkpoint metagene expression and evidence of an exhausted T cell immune response. We may hypothesize that in these tumors T-cell function is impaired by high levels of expression of immune checkpoint and it might be reactivated by treatment with ICIs. In contrast, the transcriptomic profile of the MSI immune checkpoint low group was quite similar to the MSS tumors that are in most cases resistant to ICIs. Although these tumors are a relatively better prognosis, we expect that in advanced phase of disease the T cell function is blocked by alternative mechanisms, which will not be sensitive to ICIs.

Additional data support the heterogeneity of MSI tumors. Although the majority of MSI CRC are believed to fall in the CMS1 subgroup, gene expression profiling of a series of MSI CRC revealed that 60%, 3%, 16%, and 4% of the tumors were classified as CMS1, CMS2, CMS3, and CMS4, respectively [14]. Intriguingly, it has also been reported that classification into CMS subtypes, including CMS1, may vary if the sample derives from the central tumor or from the invasive front, suggesting the possibility of intra-tumor heterogeneity [40]. This phenomenon has been confirmed by the CMS classification of a large series of 1,779 CRC from the PETACC8 clinical trial [41]. This analyses demonstrated that > 55% of CRC contain mixtures of at least two different CMSs. Intra-tumor heterogeneity was demonstrated for all the CMS groups, including CMS1. Tumors with mixed CMS1-CMS3 and CMS1-CMS4 components showed the worse disease-free survival (DFS) and overall survival (OS).

The correlation between transcriptomic heterogeneity of MSI CRC and response to ICIs has been addressed in a recent study by Gallois and collaborators [42]. Unsupervised clustering of transcriptomic data from a cohort of MSI CRC who received ICIs identified three main clusters that differentially expressed signatures that reflect the composition of the TME and the ability to proliferate of both tumor and TME cells. These signatures have been previously identified by single cell sequencing and showed association with resistance to ICIs and/or MSI status [43, 44]. The cluster A had an enrichment of signatures associated with stroma and reduced expression of signatures associated with cell cycle and immune cell infiltration. Therefore, cluster A was defined as stromal high/proliferation low. Cluster B, stromal high/proliferation medium, showed an overexpression of stromal signatures and of signatures associated with interferon-stimulated genes, infiltration of myeloid/B cells and cell cycle progression. Cluster C was characterized by low expression of stromal signatures and overexpression of cell cycle-related signatures. Importantly, cluster A was enriched of patients who had rapid progression on ICIs (30%), as compared with clusters B (12%) and C (8.1%). PFS was also longer in patients belonging to clusters B (HR 0.19) and C (HR 0.25), as compared with cluster A.

The immune heterogeneity of CRC has been recently associated with pCR in patients receiving neodjuvant anti-PD-1 therapy [45]. In particular, pCR associated with higher pre-treatment levels of CD8^+^ T cells, with low expression of PD-1 (PD-1^low^ CD8^+^), high expression of TRGC2, CD160, KLRB1 and low levels of proliferated and exhausted genes. Myeloid-derived suppressor cells (MDSCs) play a relevant role in the pathogenesis and progression of CRC and are involved in the resistance to immune therapy by suppressing T-cell activity [46, 47]. Interestingly, low levels of CD8^+^ T-cells and high levels of MDSCs in the TME have been recently correlated with resistance to PD-1 inhibitors in dMMR/MSI CRC [48]. Neutrophil leukocytes have also been shown to play an immunosuppressive activity in the TME of dMMR/MSI CRC [49]. The presence of inflammatory conditions in tumor sites and high neutrophil to lymphocyte ratio (NLR) during treatment were significantly associated with poor response to ICIs in a cohort of dMMR/MSI CRC patients [49].

The above summarized data support the hypothesis that transcriptomic analyses and at some extent immunoprofiling of the TME might identify MSI tumors resistant to ICIs. The low level of standardization of RNA sequencing and the complexity of bioinformatics analyses limit the possibility to use this approach in routine clinical practice. Genomic biomarker testing is more robust and reproducible. In this respect, it has long been demonstrated that dMMR/MSI CRC have a high TMB as consequence of the defect of the MMR DNA repair mechanism. However, significant difference in the TMB values among dMMR/MSI CRC have been described [50]. In this regard, it has been demonstrated that the TMB count is widely variable among dMMR/MSI tumors according to both types of MMR defect and site of origin. In particular, dMMR/MSI tumors with loss of MLH1/PMS2 had higher TMB values as compared with tumors showing loss of MSH2/MSH6. However, within MLH1/PMS2 deficient cancers, CRC showed higher TMB than endometrial cancer and other tumor histologies [51].

TMB variability might affect response to ICI. In a retrospective study of 22 MSI-high (MSI-H) mCRC patients treated with PD-1/PD-L1 inhibitors, Schrock and colleagues [52] found that patients with a higher TMB achieved higher response rates and longer survival as compared to those with a lower TMB. In particular, the median TMB values were 54 mutations/Mb in patients with an objective response and 29 mutations/Mb in non-responders (*P* < 0.001). In this relatively limited series, the optimal cut-off for TMB as a predictor of response to ICI in MSI CRC was in the range of 37–41 mutations/Mb. All TMB-high patients (*n* = 13) experienced an objective response, while 3/9 TMB-low patients had disease control and 6/9 experienced rapid progression. The TMB-high group treated with anti-PD-1 therapy did not reach median PFS, while the TMB-low patients showed a median PFS of 2 months. Although these findings are preliminary and must be cautiously interpreted due to the low number of patients, they suggest that TMB values might have a prognostic significance among MSI CRC patients receiving ICI and confirm the heterogeneity of MSI tumors. In agreement with this hypothesis, it has been recently reported that among 110 dMMR/MSI CRC patients receiving ICI, those with a TMB value ≤ 23 had a significantly shorter PFS (HR 4.26) and OS (HR 5.14) as compared with patients with higher TMB values [53]. Interestingly, patients receiving anti-CTLA-4 combinations showed a better PFS and OS benefit as compared with anti-PD-(L)1 monotherapy only for values of TMB > 40 mutations/Mb. However, only 30/110 patients in this analysis were treated with combinations of ICIs.

Subgroup analyses of the Keynote-177 study of pembrolizumab in MSI CRC suggested that tumors carrying *RAS* mutations did not have a PFS benefit with PD-1 blockade therapy, whereas no correlation was found with *BRAF* mutational status [19]. Interestingly, preliminary findings suggest that *RAS* mutant tumors are less inflamed as compared with *RAS* wild-type CRC [54]. However, final subgroup analyses of the Keynote-177 OS data did not show any significant difference among subgroups, including *RAS* wild-type and mutant cases [55]. Therefore, *RAS* mutational status cannot be considered as an adequate biomarker to stratify dMMR/MSI CRC patients based on current evidence.

## Conclusions and future perspectives

There is little doubt that the introduction of ICI in the treatment of dMMR/MSI mCRC has represented a major breakthrough. ICI have a significant clinical activity in a patient population with resistance to standard therapies and poor prognosis. Long lasting response to ICIs treatment have been also observed, suggesting that in selected patients this therapy might even lead to the cure of the disease. Nevertheless, the above summarized evidence suggests that dMMR/MSI CRC is a heterogeneous group of tumors, for which more appropriate therapeutic strategies are needed.

A significant proportion of dMMR/MSI mCRC are misdiagnosed. For this reason, it is highly recommended to perform screening of the MMR/MSI status through a combination of PCR- and IHC-based methods, to select correctly patients who would benefit from immunotherapy [56, 57].

It is also evident that additional biomarkers are needed to better stratify the dMMR/MSI mCRC populations with respect to the probability to respond to ICI. Although TMB assessment provides information on the intrinsic characteristics of CRC cells, characterization of the TME appears crucial to identify biomarkers of sensitivity or resistance to ICIs in dMMR/MSI CRC. As above discussed, gene expression profiling studies have indeed shown an association between expression of signatures of immune cell infiltration and sensitivity to ICIs [42]. Additional biomarkers possibly associated with resistance to ICI include the levels of CD8^+^ T-cells, the presence of MDSCs, the NLR and the immunoscore. The immunoscore, based on the quantification of CD3^+^ and CD8^+^ T cells at the tumor center and at the invasive margin, is correlated with disease-specific recurrence and OS in both MSS and MSI CRC patients [58]. The immunoscore immune-checkpoint (IC, immunoscore-IC) is a modified version scoring CD8^+^/PD-L1^+^ cells, that has shown to predict response to ICI in both lung and colorectal cancer patients [59]. In particular, in the AtezoTRIBE trial of first line FOLFOXIRI plus bevacizumab with or without atezolizumab in mCRC, the addition of atezolizumab showed a more relevant clinical activity in the immunoscore-IC high subgroup of proficient MMR (pMMR) CRC patients as compared with the low subgroup [60]. However, the predictive value of immunoscore-IC in MSI CRC needs to be evaluated. All the above described biomarkers should be assessed in clinical trials in order to identify what we could define the hot, intermediate and cold MSI CRC.

**Figure 1:**
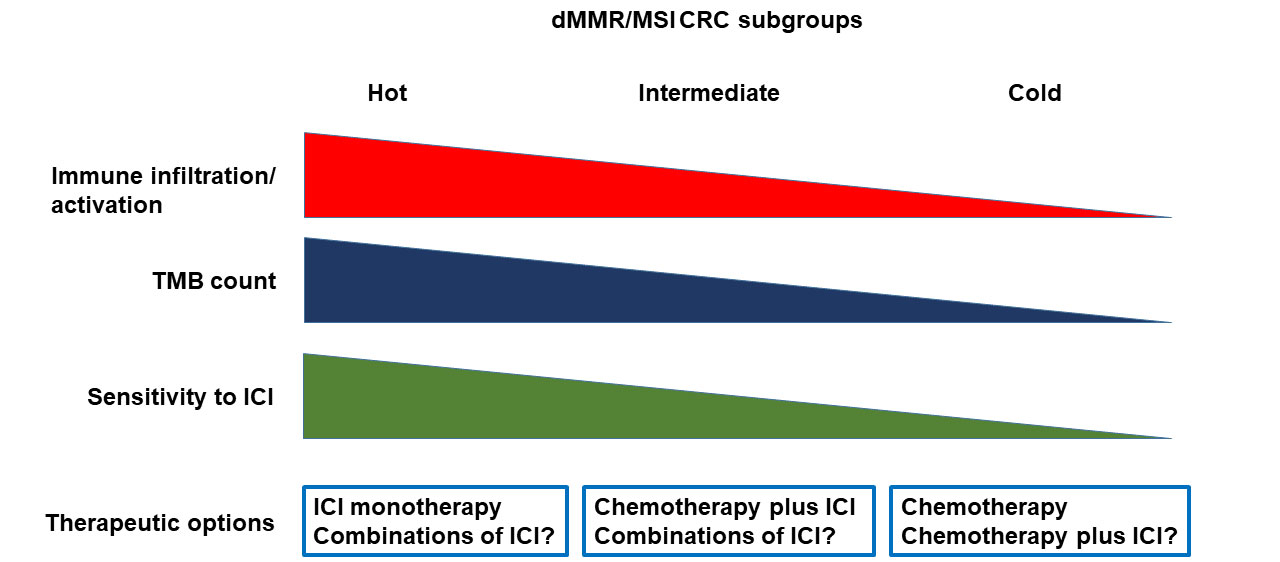
Heterogeneity of dMMR/MSI CRC and therapeutic implications. dMMR: deficient mismatch repair; MSI: microsatellite instability; CRC: colorectal carcinoma; TMB: tumor mutational burden; ICI: immune checkpoint inhibitor

The fraction of hot MSI CRC is most likely highly enriched of immune cells, recruited by the high tumor neoantigen load associated with high TMB, but at the same time impeded by the high intra-tumor levels immune checkpoint molecules (Figure 1). The relatively cold tumors may have lower levels of TMB and immune infiltrate. These tumors might have a significant intra-tumor heterogeneity with clones of CRC cells usually associated with immune exclusion or immune desert. In this fraction of MSI CRC the immune response is not likely blocked by immune checkpoint levels but by different, more complex mechanisms. Finally, the intermediate tumors may have a phenotype that is somehow in-between hot and cold MSI CRC.

While this working hypothesis needs to be formally demonstrated with appropriate experimental evidence, it does open to the possibility that different therapeutic strategies will be needed for the different MSI CRC subtypes.

The hot MSI CRC are likely to be extremely sensitive to ICIs, and ICIs as single agent might be the most appropriate therapeutic choice in these cases. Cold tumors are probably resistant to ICIs. In this subgroup, chemotherapy will be the best therapeutic approach, although combination of chemotherapy and ICI should be explored as well. The use of this combination in cold tumors is supported by results of clinical trials in pMMR/MSS CRC showing a possible synergistic activity of ICIs in combination with intensive chemotherapy regimens such as FOLFOXIRI and bevacizumab [60, 61]. In these combinations, the immunogenic cell death induced by chemotherapy is coupled with the effects of blocking the immunosuppressive activity of vascular endothelial growth factor (VEGF) [62]. Additional strategies to turn MSI cold tumors in hot tumors could be explored including: enhancing antigen presentation of dendritic cells, using DNA repair inhibitors, cytokines, or immunomodulators; increasing the activity of effector immune cells, with adoptive cell therapies and therapeutic vaccines; targeting immunosuppressive cell subsets such as MDSC; or modulating the microbiota [63].

In the intermediate tumors, the possibility that combination of ICIs might rescue response to immunotherapy in cases resistant to single agent ICIs should be explored. In particular, the combination of ipilimumab plus nivolumab appears to be associated with greater activity than the use of a single ICI in dMMR/MSI CRC [20], although no direct comparative data are available. Other ICI combinations are currently in clinical trials and the opportunity to explore them in patients with dMMR/MSI CRC will need to be evaluated based on available results. Nevertheless, the combination of chemotherapy plus ICIs might represent the best choice, in order to protect the patients from rapid progression of the disease in case of resistance to ICIs. Finally, additional therapeutic options may be based on the combination of anti-PD-1/PD-L1 antibodies with angiogenic inhibitors, which have shown to increase sensitivity to ICIs through different mechanisms, or other immunomodulating agents [64].

We do realize that the above hypothesis is highly speculative and needs validation in clinical and translational studies. We also recognize that on the basis of current knowledge it is not possible to change the approach to therapy in patients with dMMR/MSI CRC, as we do not have any validated biomarkers nor alternative therapeutic strategies available. However, evidence suggests that in the era of precision oncology the stratification of patients based on single biomarker is not able to fully recapitulate the high level of tumor heterogeneity that we face in the advanced phases of disease. More research and more biomarkers are needed to provide patients a truly personalized treatment based on their tumor characteristics.

## Abbreviations

CMS: consensus molecular subtypes
CRC: colorectal carcinoma
ctDNA: circulating tumor DNA
CTLs: cytotoxic T lymphocytes
dMMR: deficient mismatch repair
HR: hazard ratio
IC: immune-checkpoint
ICIs: immune checkpoint inhibitors
IHC: immunohistochemistry
mCRC: metastatic colorectal carcinoma
MDSCs: myeloid-derived suppressor cells
MLH1: mutL homologue 1
MMR: mismatch repair
MSH2: mutS homologue 2
MSI: microsatellite instability
MSS: microsatellite stable
OS: overall survival
pCR: pathological complete response
PCR: polymerase chain reaction
PD-1: programmed cell death 1
PD-L1: programmed cell death ligand 1
PFS: progression-free survival
PMS2: postmeiotic segregation increased 2
TMB: tumor mutational burden
TME: tumor microenvironment

## Declarations

### Copyright

© The Author(s) 2024.
